# Median Rhomboid Glossitis: A Case Study

**DOI:** 10.7759/cureus.61182

**Published:** 2024-05-27

**Authors:** Sanika R Gawre, Prasanna R Sonar, Amit Reche, Aakanksha V Tiwari, Ashtha Arya, Ayushi Singh

**Affiliations:** 1 Dentistry, Sharad Pawar Dental College and Hospital, Datta Meghe Institute of Higher Education and Research (Deemed to Be University), Wardha, IND; 2 Oral Medicine and Radiology, Sharad Pawar Dental College and Hospital, Datta Meghe Institute of Higher Education and Research (Deemed to Be University), Wardha, IND; 3 Public Health Dentistry, Sharad Pawar Dental College and Hospital, Datta Meghe Institute of Higher Education and Research (Deemed to Be University), Wardha, IND; 4 Conservative Dentistry and Endodontics, Shree Guru Gobind Singh Tricentenary Dental College, Hospital and Research Institute, Gurugram, IND; 5 Orthodontics, Sardar Patel Post Graduate Institute of Dental and Medical Sciences, Lucknow, IND

**Keywords:** tongue anomaly, antifungal therapy, tongue lesions, oral candidiasis, median rhomboid glossitis

## Abstract

A smooth, red, oval, or rhomboid patch on the dorsal midline is the classic presentation of median rhomboid glossitis (MRG), a rare and benign lesion of the tongue. MRG is still not fully understood, which presents diagnostic hurdles and calls for additional clinical investigation. It is frequently associated with candidal infections. We describe a case of a 42-year-old man who initially appeared to have either mechanical irritation or thermal injury related to a painless patch on the dorsum surface of the tongue. We document a case of MRG that was consulted for a standard dental examination in this case report. This article also highlights a dentist's need to identify the lesion and provide appropriate education for the patient.

## Introduction

Median rhomboid glossitis (MRG) affects 0.01% to 1.0% of people [1]. Males are primarily affected, with few studies indicating a gender majority [2-4]. MRG is typically found at the tongue's midline on the dorsum. It arises anterior to the circumvallate papillae and is a well-defined, symmetric, depapillated region. However, it occasionally appears in the paramedial location [5]. The lesion may have a smooth or lobular surface [1]. Although the majority of instances show no symptoms, some patients report irritation, pruritus, or chronic discomfort [1,6]. Immunosuppression in patients with MRG and a palatal inflammation known as the kissing lesion should be suspected and investigated. This has been linked to HIV/AIDS [2,7-9].

The cause of MRG remains largely unknown despite its relative prevalence [8-11]. Although the etiopathogenesis of MRG is unknown, it was previously believed to be an embryologic defect caused by the tuberculum's inability to fully unite with the tongue's lateral processes, resulting in an area of smooth, erythematous oral mucosa on the posterior dorsal surface of the tongue lacking in papillae [11-13]. These days, the majority of researchers reject this theory. According to a discovery, the oral cavity's primary source of candidal microbes is the posterior dorsal surface of the tongue. Nonetheless, certain local variables, such as surface variations in the anatomy or trauma, may facilitate the proliferation of candidal hyphae and the subsequent development of MRG [12-14]. Studies have associated several risk factors, such as diabetes mellitus, wearing dentures, and smoking, with MRG [3,15]. The most commonly recognized view holds that a significant part of the pathogenesis is caused by candidal infections [4].

It has been shown that patients frequently fail to recognize this lesion until middle age or beyond. This condition may remain undetected even after consulting with numerous practitioners. The need for a comprehensive clinical evaluation is shown by this case report, which describes the clinical presentation, diagnosis, and treatment of a patient with MRG. We hope that this case report will contribute to the existing knowledge on MRG and offer guidance on practical therapeutic approaches to this rare oral condition.

## Case presentation

**Figure 1:**
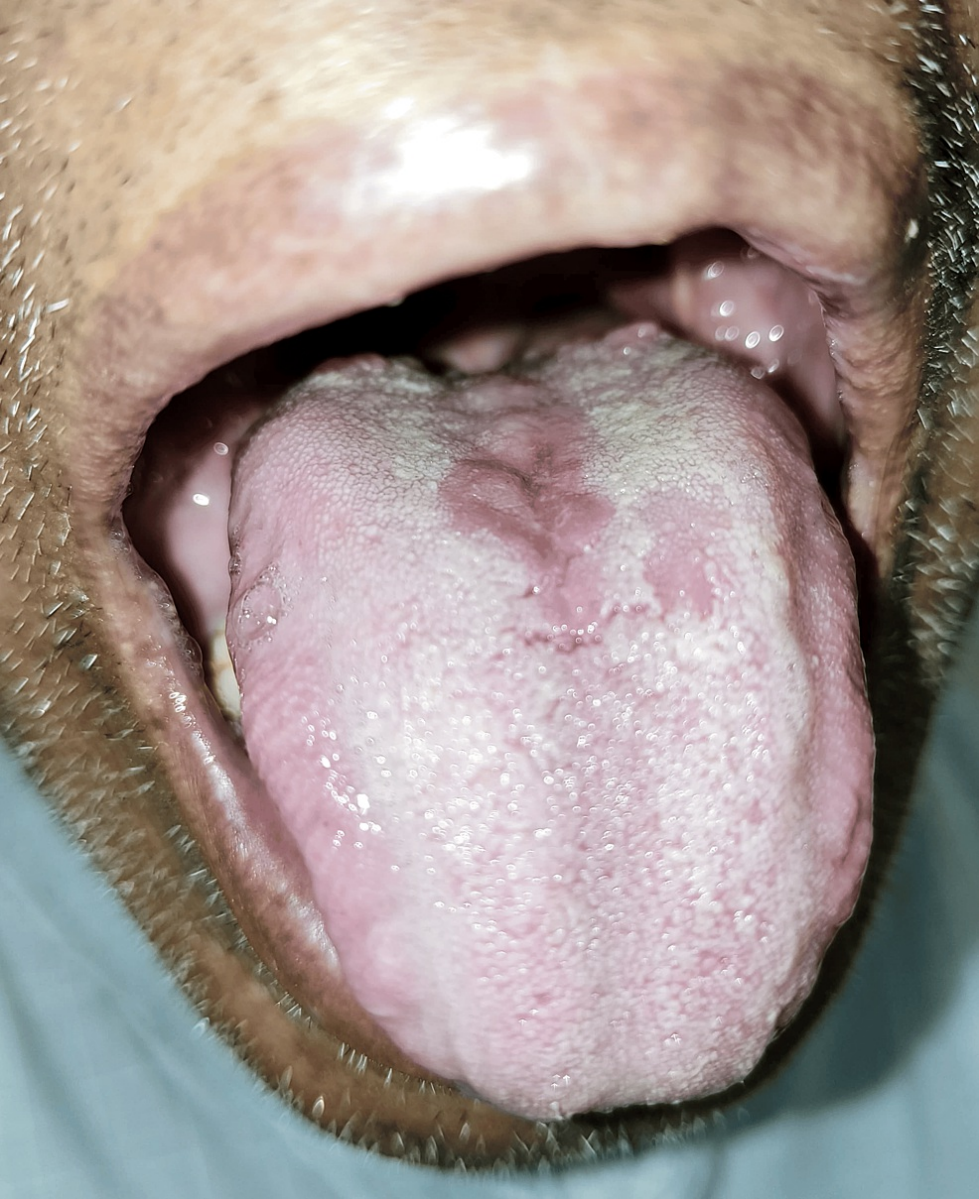
Clinical photograph of the entity. Image Credit: Aakanksha Tiwari

**Figure 2:**
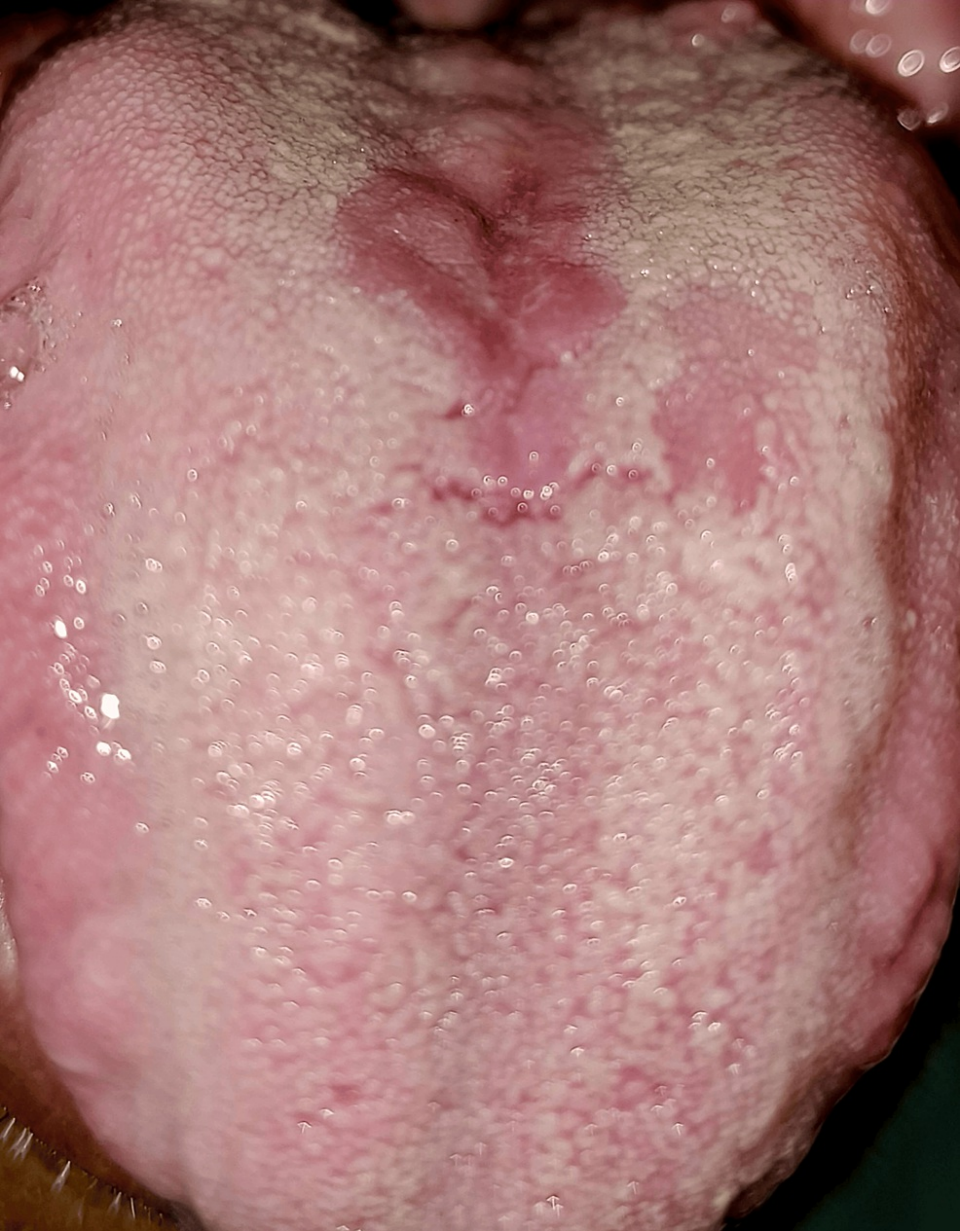
Follow-up photograph of the entity. Image Credit: Prasanna Sonar

A 42-year-old male patient arrived for his routine dental examination. A well-defined rhomboid patch of depapillation on the tongue's dorsal surface, immediately in front of the circumvallate papillae, was discovered during a clinical examination, as shown in Figure 1. The palatal mucosa was normal, and the surface was elevated and smooth. History showed that it had been constant since early childhood. The medical history was not significant. MRG was the tentative diagnosis after reviewing the medical history and clinical examination. To rule out candidiasis as the potential cause of MRG, the patient received 50 mg of fluconazole once daily for 14 days; however, the lesion failed to fade away. MRG was the clinical diagnosis made. Since it was asymptomatic, no medical intervention was suggested. Generally, MRG does not require extensive investigation unless there are atypical features or symptoms suggesting a more systemic involvement. After three months, he felt reassured and was informed that regular monitoring was required. Figure 2 shows a follow-up photograph of the patient after three months.

## Discussion

Many tongue lesions raise health concerns for patients and professionals equally. As a result, knowledge of the genesis, clinical manifestations, diagnosis, and treatment of these disorders is essential for dentists. While the frequency of MRG varied from 0.9% to 5.4% in prior research, a previous study found that the prevalence rate was 0.2% [3]. Men are impacted three times more frequently than women, according to Rogers and Bruce [2]. However, among 28 MRG patients, Wright [4] found a 4:1 female prevalence. A study discovered that among 12 MRG patients, the female-to-male ratio was 11:1 [3].

Even though *Candida* has been linked to MRG, its precise cause is yet unknown [1,4,8,9,16]. Research might concentrate on examining additional potential infectious agents and verifying the etiological involvement of *Candida*. Research investigating potential environmental, immunological, and genetic triggers for the development of MRG can be conducted. There is disagreement concerning the precise cause of MRG. Several factors, including wearing dentures, smoking, having diabetes, using corticosteroids as sprays or inhalers, and having HIV, have been suggested as potential causes [16]. According to Joseph and Savage [1], individuals receiving broad-spectrum antibiotics, diabetics, and immunosuppressed patients have a higher prevalence of MRG. Currently, the majority of studies concur that MRG is linked to candidal infections. According to the researchers, MRG is one of the most common candidal infections in people with diabetes mellitus [17]. Research by Ghabanchi et al. on Iranian patients with diabetes revealed a higher prevalence of MRG compared to the control group [18]. According to a study by Deshpande and Bharucha, a 60-year-old man had a rare case of MRG with significant colonies of actinomycosis [19]. According to certain research, oral candidiasis appears to be significantly predisposed to be caused by smoking, prostheses, and minor traumas, either alone or in combination with one another [5,20]. According to Gümrü et al. [21], denture stomatitis and MRG are frequently associated. Arendorf and Walker evaluated the role that smoking and wearing dentures had in the development of MRG. A significant percentage of MRG patients also smoked tobacco and wore dentures; these local variables may have contributed to the genesis of MRG [22].

According to Arendorf and Walker [22], the tongue is the main reservoir for *Candida*, and 44% of people have these organisms as a typical part of their oral flora. Specifically, the middle region of the tongue is an ideal location for organisms to proliferate intensely. Whitaker and Singh [23] have established a close relation between the tongue's surface that touches the palate and where MRG develops because the tongue and palatal mucosa remain in close contact during swallowing and when the tongue is at rest. Furthermore, Farman suggests that the tongue may be more susceptible to candidiasis and the subsequent loss of filiform papillae if there is reduced blood flow [24]. The coexistence of MRG with palatal inflammation is referred to as a "kissing lesion" due to the inflammation indicating contact with the affected region of the tongue. Considering the established correlation between immunosuppression and AIDS, suspicions should be raised [2]. Therefore, these data may suggest a prolonged contact between the hard palate and the midline dorsum of the tongue, which is infected with *Candida*, leading to severe lesions.

MRG lesions often exhibit asymptomatic characteristics. Similarly, in our case, the patient showed no symptoms. Consequently, the patient did not require therapy; however, individuals of this type have been monitored. The afflicted area can be treated directly with topical antifungals such as clotrimazole or nystatin. Fluconazole and other systemic antifungal medications may be administered for more severe instances. It is crucial to keep the oral cavity healthy, and it is important to address underlying systemic issues [6,19-21]. It is recommended for every individual with a kissing lesion to be screened for HIV, as the presence of lesions on the palate may indicate potential HIV infection. Despite the claims that there is no apparent association between MRG and cancer [8], reports of MRG's malignant transformation exist [25,26]. We recommend performing a biopsy to rule out malignant change, particularly if the lesion appears to be ulcerated or solid to the touch.

## Conclusions

MRG continues to raise concerns about its significance and pathogenesis. Previous studies have demonstrated a substantial correlation between MRG presence and systemic illnesses. A developmental condition of the tongue that is often unrecognized and asymptomatic is called MRG. This lesion frequently remains undiagnosed until the middle years of life. A thorough understanding of tongue diseases is necessary to make an accurate diagnosis. Therefore, the role of healthcare providers, such as dentists, is crucial, as they are primarily responsible for working with the oral cavity and can identify the disease during the patient's initial oral examination. A biopsy is suggested if there is uncertainty about the type of lesion. Antifungal therapy is recommended for lesions that exhibit symptoms; however, in cases where the lesion is asymptomatic, no treatment is required. Clinicians may prescribe systemic antifungal medications such as fluconazole as well as topical alternatives such as nystatin or clotrimazole. Finally, the reported case with MRG emphasizes how crucial it is to take this diagnosis into account when central papillary atrophy on the tongue is observed. This has important implications for both diagnosis and treatment. It is necessary to do additional research to better understand the pathophysiological mechanisms and etiological factors underlying MRG in order to improve therapy and diagnostic precision. By keeping track of these cases and studying them further, we can improve our knowledge and treatment of this unusual but noteworthy oral ailment.

## References

[1] Joseph BK, Savage NW (2000). Tongue pathology. Clin Dermatol.

[2] Rogers RS 3rd, Bruce AJ (2004). The tongue in clinical diagnosis. J Eur Acad Dermatol Venereol.

[3] Avcu N, Kanli A (2003). The prevalence of tongue lesions in 5150 Turkish dental outpatients. Oral Dis.

[4] Wright BA (1978). Median rhomboid glossitis: not a misnomer: review of the literature and histologic study of twenty-eight cases. Oral Surg Oral Med Oral Pathol.

[5] Lago-Méndez L, Blanco-Carrión A, Diniz-Freitas M, Gándara-Vila P, García-García A, Gándara-Rey JM (2005). Rhomboid glossitis in atypical location: case report and differential diagnosis. Med Oral Patol Oral Cir Bucal.

[6] Carter LC (1990). Median rhomboid glossitis: review of a puzzling entity. Compendium.

[7] McNally MA, Langlais RP (1996). Conditions peculiar to the tongue. Dermatol Clin.

[8] Goregen M, Miloglu O, Buyukkurt MC, Caglayan F, Aktas AE (2011). Median rhomboid glossitis: a clinical and microbiological study. Eur J Dent.

[9] Panta P, Erugula SR (2015). Median rhomboid glossitis-developmental or candidal?. Pan Afr Med J.

[10] van der Wal N, van der Kwast WA, van der Waal I (1986). Median rhomboid glossitis. A follow-up study of 16 patients. J Oral Med.

[11] Soysa NS, Ellepola AN (2005). The impact of cigarette/tobacco smoking on oral candidosis: an overview. Oral Dis.

[12] Terai H, Shimahara M (2007). Partial atrophic tongue other than median rhomboid glossitis. Clin Exp Dermatol.

[13] Yarom N, Cantony U, Gorsky M (2004). Prevalence of fissured tongue, geographic tongue and median rhomboid glossitis among Israeli adults of different ethnic origins. Dermatology.

[14] Espinoza I, Rojas R, Aranda W, Gamonal J (2003). Prevalence of oral mucosal lesions in elderly people in Santiago, Chile. J Oral Pathol Med.

[15] Bánóczy J, Rigó O, Albrecht M (1993). Prevalence study of tongue lesions in a Hungarian population. Community Dent Oral Epidemiol.

[16] Samaranayake LP, Keung Leung W, Jin L (2009). Oral mucosal fungal infections. Periodontol 2000.

[17] Guggenheimer J, Moore PA, Rossie K (2000). Insulin-dependent diabetes mellitus and oral soft tissue pathologies. I. Prevalence and characteristics of non-candidal lesions. Oral Surg Oral Med Oral Pathol Oral Radiol Endod.

[18] Ghabanchi J, Andisheh Tadbir A, Darafshi R, Sadegholvad M (2011). The prevalence of median rhomboid glossitis in diabetic patients: a case-control study. Iran Red Crescent Med J.

[19] Deshpande RB, Bharucha MA (1991). Median rhomboid glossitis: secondary to colonisation of the tongue by Actinomyces (a case report). J Postgrad Med.

[20] Woods TR, White J, Koutlas I (2023). Fungal lesions of the oral mucosa diagnosis and management. Oral Maxillofac Surg Clin North Am.

[21] Gümrü B, Kadir T, Uygun-Can B, Ozbayrak S (2006). Distribution and phospholipase activity of Candida species in different denture stomatitis types. Mycopathologia.

[22] Arendorf TM, Walker DM The prevalence and intra-oral distribution of Candida albicans in man. Arch Oral Biol.

[23] Whitaker SB, Singh BB (1996). Cause of median rhomboid glossitis. Oral Surg Oral Med Oral Pathol Oral Radiol Endod.

[24] Farman AG (1976). Atrophic lesions of the tongue: a prevalence study among 175 diabetic patients. J Oral Pathol.

[25] Sharp GS, Bullock WK (1958). Carcinoma arising in glossitis rhombica mediana. Cancer.

[26] Burkes EJ, Lewis JR (1976). Carcinoma arising in the area of median rhomboid glossitis. Oral Surg Oral Med Oral Pathol.

